# Reducing Alarm Burden in a Level IV Neonatal Intensive Care Unit

**DOI:** 10.1097/pq9.0000000000000386

**Published:** 2021-02-12

**Authors:** Kortany E. McCauley, Alissa A. Schroeder, Tawney K. DeBoth, Alexander M. Wiebe, Christopher L. Bosley, Diane D. Ballweg, Jennifer L. Fang

**Affiliations:** From the * Division of Neonatal Medicine, Mayo Clinic, Rochester, Minn.; † Department of Nursing, Mayo Clinic, Rochester, Minn.; ‡ Mayo Clinic Healthcare Technology Management, Rochester, Minn.; § Department of Anesthesiology, Mayo Clinic, Rochester, Minn.

## Abstract

**Introduction:**

Excessive alarm burden contributes to alarm fatigue, causing staff to ignore or delay response to clinically significant alarms. The objective of this quality improvement project was to reduce yellow self-resolving SpO2 alarms from a mean of 14 alarms/patient-hour (APH) to 7 APH (a 50% reduction) within a 6-month period, without significantly decreasing the amount of time spent in target SpO2 range (90%–95%).

**Methods:**

A multidisciplinary team used Define-Measure-Analyze-Improve-Control methodology to identify etiologies of alarm frequency and design improvement interventions to reduce alarm burden in a single-site Level IV NICU. Data-driven changes in alarm limit settings, alarm delay, and trial of a new pulse oximeter probe were used. Alarm data from the bedside monitor were analyzed following each improvement cycle. As a balancing measure, histograms monitored time spent in target SpO2 range.

**Results:**

SpO2 alarm data were collected for 4,320 patient-hours (180 patient-days) on 40 neonatal intensive care unit patients meeting inclusion criteria. Corresponding histograms were obtained for each patient day. Following 5 Plan-Do-Study-Act cycles, the mean number of yellow self-resolving SpO2 alarms decreased from 14 to 5 APH, a 64% decrease. There was no difference in time spent in target SpO2 range (50% versus 50%, *P* = 0.93). After achieving the project aim, 2 control phase measurements demonstrated sustained improvement (mean APH = 6).

**Conclusions:**

Yellow self-resolving SpO2 alarm frequency was reduced by 64% through the implementation of data-driven changes in alarm limit settings, alarm delays, and trial of a more sensitive oximeter probe without introducing harm to patients.

## INTRODUCTION

As the number of monitored physiological parameters expands in intensive care units, alarm frequency and exposure increase. Alarm fatigue, defined as a condition of sensory overload for staff exposed to an excessive number of alarms,^1^ also grows. Alarm fatigue is a well-recognized and an important safety concern,^2–7^ as evidenced by the Joint Commission issuing a Sentinel Event Alert in 2013 that identified alarm fatigue as a national safety concern.^8,9^ Excessive alarm burden may result in clinicians ignoring, silencing, or delaying response to clinically significant alarms leading to an unsafe care environment.^10–13^ The neonatal intensive care unit (NICU) is a technology-resourced care setting with various alarm sources, including ventilators, infusion pumps, incubators, and vital sign (VS) monitors. Previous reports have shown alarm burden and alarm fatigue are problematic in the NICU.^2,5,12,14–16^

Alarm fatigue was identified as a challenge in our NICU, so a multidisciplinary quality improvement (QI) team was assembled to address this issue. The goal of this QI effort was to identify the largest contributors to alarm frequency and implement alarm management strategies to reduce alarm burden. Using the Define-Measure-Analyze- Improve-Control methodology, we defined our quality gap, measured baseline performance, and identified key causes for the gap that informed tests of change.

After direct observation, we determined VS monitors were the most frequent alarm source. By analyzing data from the VS monitors, we were able to specifically identify yellow (advisory) self-resolving oxygen saturation (SpO_2_) alarms as the greatest contributor to alarm burden. The QI team developed a specific aim to reduce yellow self-resolving SpO_2_ alarms from a mean of 14 alarms/patient-hour (APH) to 7 APH (a 50% reduction) within a 6-month period, without significantly decreasing the amount of time spent in target SpO_2_ range (90%–95%). The purpose of this report is to share our experience and lessons learned with other care teams striving to reduce alarm burden within their intensive care settings.

## METHODS

### Context

The project was carried out at a single-site, 34-bed level IV NICU at Mayo Clinic in Rochester, Minn. The NICU consists of 20 patient beds in open-bay rooms, 6 beds in semiprivate rooms, and 8 beds in private rooms. More than 350 infants receive care in the level IV NICU annually, with 18% being very low birth weight.

Each NICU bed is equipped with a patient monitor (IntelliVue MP70, Philips Healthcare, Andover, Mass.). From admission through discharge, the monitor acquires, digitizes, and displays physiological data; monitors alarm conditions; and issues alarm notifications.^17^ The Philips patient monitoring system classifies alarms as yellow (advisory) and red (critical).^15,17^ Yellow alarms are low-priority alarms that call attention to alarm limit (threshold) violations not considered acutely life-threatening. Red alarms are high priority alarms and indicate the potential presence of a life-threatening condition or critical device failure. The default numeric specifications for yellow and red alarms were determined by our clinical practice and set by Mayo Clinic Healthcare Technology Management. When indicated, alarm specifications could be changed by clinicians to suit the clinical needs of patients.

Patients receiving titratable supplemental oxygen were included in this QI project. Patients with congenital heart disease, congenital diaphragmatic hernia, pulmonary hypertension, or both preductal and postductal saturation monitoring were excluded. The SpO_2_ target for included patients was 90%–95%. Before our improvement efforts, the default red SpO_2_ alarm limit was <80% with an averaging time of 20 seconds. Default yellow SpO_2_ alarm limits were <90% and >95% with an averaging time of 10 seconds. An audible alarm would sound if the SpO_2_ value breached the high or low alarm limits on the monitor for >10 seconds (termed alarm delay). Yellow SpO_2_ alarms could self-resolve (silence automatically if a patient no longer exceeded the alarm limit) or could be manually silenced by staff.

Our multidisciplinary QI team included physicians, staff nurses, advanced practice nurses, respiratory therapists, and an Healthcare Technology Management-Healthcare Engineering Technician familiar with Philips monitor technology.

### Defining the Problem and Planning the Interventions

Following 10 hours of direct observation during which alarm-producing sources were tallied, the VS monitor was determined as the greatest source of audible alarms [75% (564/751) all audible alarms], followed by ventilator, bed alarm, IV pump, and others.

By downloading, categorizing, and analyzing all VS alarm data, that is, HR, RR, or SpO_2_, we found SpO_2_ alarms were most frequent. SpO_2_ alarm data were categorized as red (6%) or yellow (94%). Yellow alarms were further categorized as silenced (33%) or self-resolving (67%). Thus, yellow self-resolving SpO_2_ alarms were identified as most frequent.

For each alarm, the Philips monitor archived a time stamp, the alarm condition (HR, SpO_2_, etc.), and the associated alarm priority (red or yellow). Alarm messages also contained the value of the threshold, frequency, and action. For example, a patient may have an archived alarm at 12:30 am for SpO_2_ (condition) of 84% lasting >10 seconds (threshold), yellow (alarm priority), and silenced by staff.

Although archived data identify whether the alarm was paused, silenced, or self-resolved, it was unclear whether self-resolving alarms were associated with staff intervention (eg, increasing the FiO_2_ or repositioning the patient) or if the patient’s SpO_2_ returned to target range without intervention. To determine if yellow SpO_2_ self-resolving alarms were associated with nonintervention (hence, nuisance alarms), we performed direct observation in the open-bay rooms. Three team members spent 1 hour in the NICU observing staff response to yellow SpO_2_ alarms. During this time, 179 yellow SpO_2_ alarms were documented. Of these, 131 were self-resolved and 48 were silenced. We found 98% (128/131) of the yellow self-resolving alarms required no intervention. Conversely, 50% (24/48) of the yellow silenced alarms required intervention. Yellow self-resolving SpO_2_ alarms were significantly correlated with nonintervention (*P* < 0.05).

Thus, yellow self-resolving SpO_2_ alarms were largely considered nuisance alarms and targeted as our primary improvement measure.

**Fig. 1.**
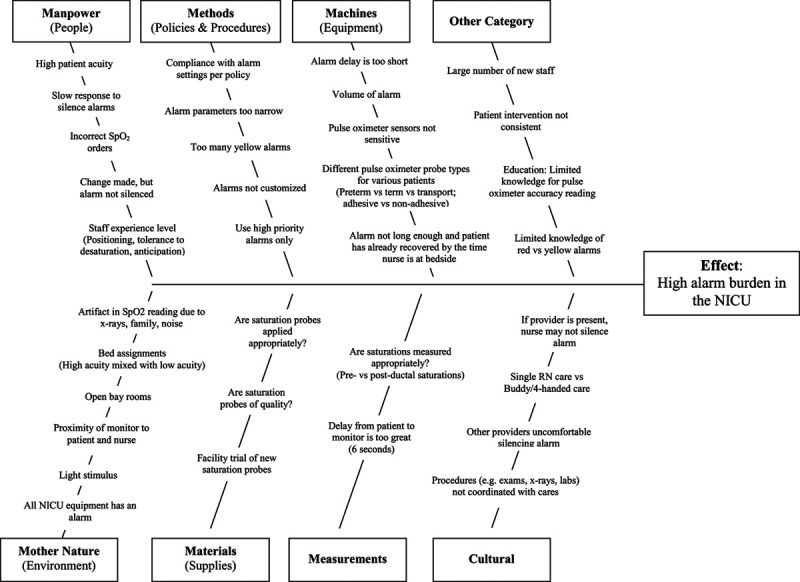
Fishbone Diagram of contributors to high alarm burden in the NICU.

**Fig. 2.**
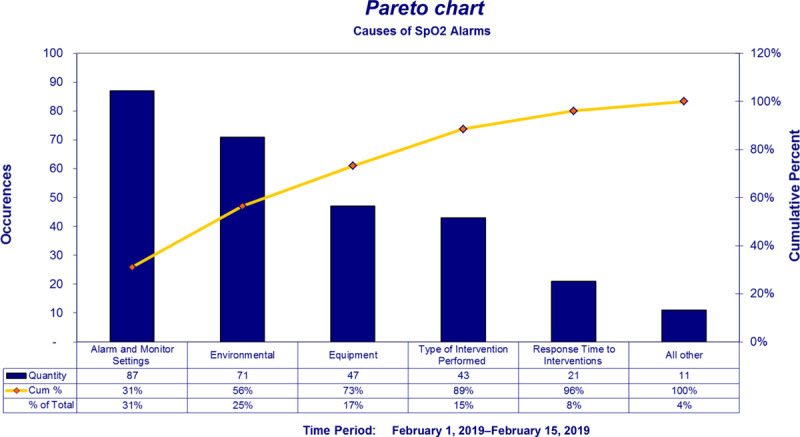
Pareto chart of causes of SpO_2_ alarms (from preintervention survey).

During a brainstorming session, QI team members identified key causes of yellow self-resolving SpO_2_ alarm frequency and summarized results in a fishbone diagram (Fig. 1). To further assess the scope of the problem and prioritize key causes of alarm burden, a preintervention electronic survey (REDCap) was sent to NICU staff. The survey was developed by our multidisciplinary team and informed by our initial brainstorming session and unit culture. Survey questions about impact on patient care, trust in alarms, and patient safety were on a 1–5 Likert Scale (1 = strongly agree, 5 = strongly disagree). Respondents answered “yes” or “no” when asked about witnessing a delay in response to an urgent situation due to alarm fatigue, witnessing patient harm as a result of alarm fatigue, or having parents express concerns about the promptness of staff to respond to alarms. Respondents also ranked what they perceived to be the top three contributors to SpO2 alarm frequency. Survey data were used to create a Pareto chart, which indicated VS “alarm and monitor settings” were the greatest contributor (Fig. 2). Monitor settings, alarm limits, and pulse oximeter technology were then selected as targets for improvement interventions.

### Intervention

Based on modifiable VS monitor settings and review of other QI initiatives focused on alarm burden, we identified two monitor settings that could be changed during our Plan-Do-Study-Act (PDSA) cycles: (1) SpO_2_ alarm limit settings and (2) alarm delay. During this QI initiative, Mayo Clinic trialed a new pulse oximeter (Masimo and is RD SET Specialty Sensor Series, Calif.^18^) within various critical care settings, including our NICU. This was incorporated as a third test of change.

Each PDSA cycle included 30 patient-days (n = 30, 720 patient-hours) and lasted approximately 2–4 weeks depending on the number of patients meeting inclusion criteria. Some patients were sampled more than once during a PDSA cycle, but these patients were sampled on different days and limited to 3 data points per cycle. Although different alarm settings were being tested, the target SpO_2_ range (90%–95%) remained unchanged throughout each cycle and was communicated to staff at each education point. During each PDSA cycle, QI team members periodically verified alarm limit accuracy by visibly checking the bedside monitor at the start of a randomly selected shift. PDSA cycle interventions are summarized in Table 1.

**Table 1.** Summary of PDSA Interventions

PDSA Cycle	Date (2018/2019)	SpO2 Alarm Limits (%)	New SpO_2_ Sensor Probe (Y/N)	Lower SpO_2_ Alarm Delay (s)	Higher SpO_2_ Alarm Delay (s)	Alarms per Patient-hour (Mean)	Percent Time SpO2 in Target Range (90%–95%)
Baseline	November–December	90–95	N	10	10	14	50
1	March	88–97	N	10	10	10	47
2	April	88–97	Y	10	10	8	54
3	May	88–97	N	20	10	6	49
4	May–June	88–97	Y	20	10	8	51
5	July	86–97	Y	20	20	5	50
Control Phase 1	October–November	86–97	N	20	20	6	N/A
Control Phase 2	November–December	86–97	N	20	20	6	N/A

N, no; N/A, not applicable; Y, yes.

1. The first PDSA cycle reduced the lower alarm limit from 90% to 88% and increased the upper alarm limit from 95% to 97%, thus expanding the alarm limit settings from 90%–95% to 88%–97%.
2. The second PDSA cycle continued the 88%–97% alarm limits and incorporated the trial of the Masimo RD SET Specialty Sensor Series^18^ pulse oximeter sensor (the Masimo SET^19^ was used at baseline). The nurse quality coach, respiratory therapist, and Masimo Key Account Clinical Specialist were present during the installation of the new oximeter sensors to ensure appropriate placement.
3. The third PDSA cycle increased the low-limit yellow alarm delay from 10 to 20 seconds. Lengthening the alarm delay allowed for short-term SpO_2_ fluctuations without triggering alarms. We thought staff would be less responsive to high saturations, so only the low-limit alarm delay was changed during this PDSA cycle. Because trial of the new Masimo RD SET Specialty Sensor^18^ had ended, the baseline Masimo SET^19^ oximeter sensor was reinstituted.
4. The fourth PDSA cycle implemented all previous PDSA cycle changes including alarm limit settings of 88%–97%, use of the new trial Masimo RD SET Specialty Sensor,^18^ and SpO_2_ low-limit alarm delay of 20 seconds.
5. The fifth PDSA cycle reduced the lower SpO_2_ alarm limit to 86% (broadening limits to 86%–97%) and increased the high SpO_2_ yellow alarm delay from 10 to 20 seconds. The lower SpO_2_ alarm delay of 20 seconds and use of Masimo RD SET Specialty Sensor^18^ was continued. Alarm delays were hard-coded as defaults in the monitor.

### Study of the Intervention

**Fig. 3.**
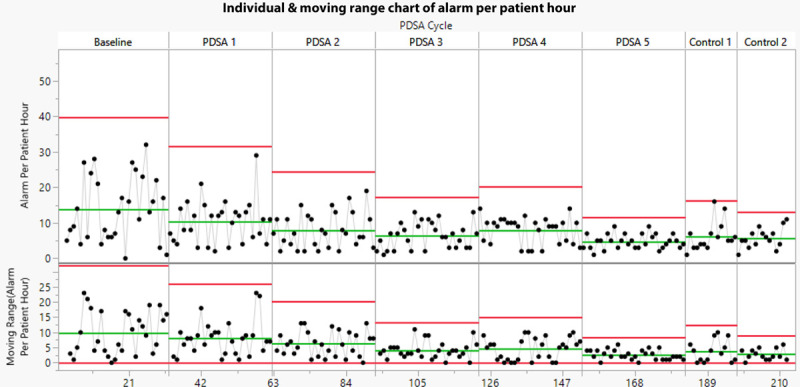
Control chart of yellow (SpO2) self-resolving alarms per patient-hour. Baseline mean of 14 APH to 5 APH after PDSA Cycle 5. Two control phases (phase 1 and phase 2) with mean of 6 APH.

After each PDSA cycle, 24 hours of data (07:00 to 07:00) was downloaded from the VS monitor for 30 sequential NICU patients meeting inclusion criteria (for a total of 720 patient-hours). Alarm limit settings for that 24-hour period were verified from the monitor data to confirm nursing compliance with the correct alarm limit settings. Once verified, the yellow self-resolving SpO_2_ APH value was calculated and plotted on an individual and moving range control chart (Fig. 3). The overall mean APH for the PDSA cycle was determined.

### Measures and Analysis

Our primary improvement measure was yellow self-resolving SpO_2_ APH, calculated using downloaded archived data from the Philips monitor. This was chosen because it was a reliable, automatic data source. Secondary improvement measures included staff perception of alarm fatigue, willingness to address high alarm burden, and improved patient safety as measured via survey.

Our balancing measure was time in the target SpO_2_ range (90%–95%), as we did not want interventions to significantly decrease the amount of time patients spent in target range. The Philips monitor archives this data in the form of histograms for each individual patient. Histograms were collected for each patient at baseline and throughout each PDSA cycle. Each histogram represented 24 hours (1 patient day) of SpO_2_ data; hence, 30 patient-days of histogram data were acquired each cycle. The percent of time spent in range was calculated for each patient and averaged after each PDSA cycle.

During the improvement phase, data were plotted in time series on the individual and moving range control chart (Fig. 3). The balancing measure at baseline and after the improvement interventions was compared using a two-tailed *t* test. Comparison of preintervention and postintervention staff survey results were compared using the Pearson chi-square test. All data analyses were conducted using JMP 14.1.0 SAS Institute Software.

The Institutional Review Board deemed this project exempt from review as activities (alarms) rather than human subjects was the focus, thereby not meeting the definition of human subjects’ research requiring Institutional Review Board approval.

## RESULTS

Between November 2018 and July 2019, yellow self-resolving SpO_2_ alarm data were collected for 4,320 patient-hours (180 patient-days) on 40 NICU patients meeting inclusion criteria. Corresponding histograms were obtained for each patient day (100%). Cohort mean gestational age was 27 weeks 3 days (SD 4 days), and mean birthweight was 1,097 g (SD 629 g). Eighty-five percent of patients were very low birth weight (birth weight < 1500 g). Of the 180 patient-days, 63% (n = 113) were on continuous positive airway pressure, 31% (n = 55) on mechanical ventilation, 6% (n = 10) on high flow nasal cannula, and 1% (n = 2) on noninvasive positive pressure ventilation.

After 5 sequential PDSA cycles, the mean number of yellow self-resolving SpO2 APH decreased from 14 to 6, a 64% decrease (Fig. 3; Table 1). A reduction in mean APH was demonstrated following each PDSA cycle except cycle 4. The variation in the number yellow self-resolving alarms was also reduced as illustrated in the Moving Range chart and the lowering of the upper control limit.

To maintain compliance with the practice changes that resulted from this QI initiative, default settings for SpO2 alarm parameters on the bedside monitors were hard-wired and automatically set to 86%–97%. For the project control plan, NICU quality coaches collected and analyzed yellow self-resolving SpO2 data (n = 15) for the first month of each quarter. Two control phase measurements were conducted, and both demonstrated a mean of 6 APH—showing sustained improvement in our primary measure. For future control phase measurements, an action plan will be initiated if APH is ≥ 10 for two consecutive quarters, which includes: (1) charge nurse verification of accurate alarm limit settings before each shift for 5 days and (2) alarm data review at the institutional Neonatal Quality and Safety Committee where next steps would be determined.

Throughout the QI initiative, the balancing measure was monitored. At baseline, patients spent an average of 50% of each 24-hour period within the target SpO_2_ range. Throughout the PDSA cycles, there was no significant change in percent time spent in target range (Table 1). During the final PDSA cycle, patients spent an average of 50% time within target, which was not significantly different from baseline (*P* = 0.93). The time our patients spent within the target SpO_2_ range is similar to other studies that reported percent time in range of 47%–54%.^20–22^

The preliminary staff survey had a response rate of 50% (115/232) as did the postintervention survey (117/232) (Table 2). Following the improvement interventions, significantly fewer staff reported parent concerns about promptness of staff responding to alarms (51% versus 32%, *P* < 0.01). In addition, fewer staff reported the need to customize SpO_2_ alarm limits or modify SpO_2_ target (52% versus 39%, *P* = 0.04). There were no significant differences in the responses to other survey questions.

**Table 2.** Staff SURVEY

Survey Question	Baseline (n = 115) (% Agree)	Postintervention (n = 117) (% Agree)	*P*
Alarms disrupt patient care	83	85	0.33
Frequent alarms reduce trust in alarms and cause caregivers to inappropriately turn off or ignore alarms	84	91	0.38
Witnessed a delay in response to an urgent situation due to alarm fatigue	37	34	0.64
Witnessed patient harm as a result of alarm fatigue	9	3	0.09
Parents express concerns about the promptness of staff to respond to alarms	51	32	< 0.01
Customize SpO_2_ alarm limits or modify SpO_2_ target order	52	39	0.04
Address alarm fatigue and/or offer help to remedy the situation	78	90	0.12

## DISCUSSION

This QI initiative successfully reduced yellow SpO_2_ self-resolving alarms by 64%, surpassing the project aim. Small data-driven changes in high and low alarm limit settings, alarm delay, and use of a new pulse oximeter probe were used to achieve success in alarm reduction. Decreased alarm frequency has persisted as 2 subsequent control measures demonstrated a mean of 6 APH. The balancing measure of time spent within the target SpO_2_ range was not adversely affected. Survey results suggested there was less need for staff to modify alarm limits following our improvement interventions, and parental concern about staff responsiveness to alarms was reduced.

Changes in alarm limit settings and alarm delays have demonstrated efficacy in other QI initiatives aimed at reducing alarm frequency. Johnson et al^23^ employed similar strategies by reducing the low oximeter alarm limit and increasing low alarm delay which accomplished a 78% reduction in the mean number of nonactionable low alarms. Patient-specific alarm limit parameters based on the patient condition or postmenstrual-age profiles have also been successful in reducing alarm frequency.^23–27^

Although previous publications used direct observation and tallying methods,^23^ this QI initiative utilized objective patient data archived within each individual bedside monitor. Our use of direct data downloads reduced the risk of human error and allowed for accurate, consistent measurement of our primary improvement and balancing measures. Once improvement in yellow self-resolving SpO2 alarm frequency was achieved, the new alarm limit settings and alarm delays were hard-wired into the VS monitor enhancing standardization and compliance with practice changes. Utilizing the automatic data reporting capabilities of the bedside VS monitor to measure, reduce, and maintain decreased alarm burden in an NICU is unique to this QI initiative.

We also tested and implemented a new pulse oximeter probe, which has not been reported in other studies aimed at reducing alarm frequency in the ICU setting. Equipment was thought to represent the third leading cause of SpO_2_ alarms in our unit (Fig. 2). Because Mayo Clinic was trialing a new pulse oximeter probe in the ICUs, this trial was included as a PDSA cycle (Cycle 2). The pulse oximeter probe may have facilitated more consistent and accurate SpO_2_ readings, thereby reducing false alarms. This finding highlights the important consideration of how medical equipment impacts alarm frequency, that is, the “Machines” category of a Fishbone diagram.

Strengths of this project include the collaborative effort among the multidisciplinary team. Before the QI team, nursing staff attempted to address alarm fatigue independently as excessive alarm burden was considered by many to be primarily a nursing issue. After engaging skill sets from various team members, strategies to successfully reduce alarm frequency were developed and tested. The ability to download alarm data from each patient monitor improved the validity of this project as counts were automatically generated and independent of human tallies. Furthermore, continuous SpO2 data analysis via histograms provided ongoing assessment of the safety of interventions.

This QI project is not without limitations. First, the setting for the project occurred in a Level IV NICU that consists primarily of open-bay rooms and some semiprivate and private rooms. Therefore, outcomes for this project may not translate as well for single-room units with escalating alarm or paging systems. Another potential limitation is that some patients were sampled more than once to maintain the pace of improvements. To minimize the effect of repeated sampling, the patient SpO2 data were downloaded on different days within a PDSA cycle and limited to 3 data points per cycle. Although these limitations may reduce applicability to other units, the project cohort provided an accurate representation of the population in our NICU as patients were of different gestational ages, postmenstrual ages, and on various respiratory supports.

## CONCLUSIONS

Alarm fatigue is a well-recognized and important safety concern. In hospital settings where numerous physiological parameters are monitored by technology, alarm fatigue as a result of high alarm frequency is commonplace. This report provides a roadmap for how yellow self-resolving SpO2 alarm frequency was successfully reduced by 64%. These improvements were achieved by changes in alarm limit settings, alarm delays, and trial of a more sensitive pulse oximeter probe without introducing harm to patients. Expanding similar methods to additional alarm sources such as other physiologic parameters measured by the bedside monitor or other medical equipment (ie, infusion pumps, incubators, and ventilators) may further reduce alarm frequency in the ICU setting.

## DISCLOSURE

The authors have no financial interest to declare in relation to the content of this article.

## Acknowledgments

We thank Leslie Moody, BSN, Leah Heidenreich, MD, Gloria Akuamoah-Boateng, MB, and Hannah Giunta, DO, PhD, for study design contributions and data collection, Mandy Reckward, APRN and Melanie Glynn, APRN for study design contributions and communication coordination with staff, and Nanette Matzke, LRT for assistance with intervention implementation and patient safety.
